# Influence of electron density spatial distribution and X‐ray beam quality during CT simulation on dose calculation accuracy

**DOI:** 10.1120/jacmp.v12i3.3432

**Published:** 2011-04-06

**Authors:** Ahmad Nobah, Belal Moftah, Nada Tomic, Slobodan Devic

**Affiliations:** ^1^ Biomedical Physics Department King Faisal Specialist Hospital and Research Center Riyadh Saudi Arabia; ^2^ Department of Radiation Oncology Jewish General Hospital, McGill University Montréal Québec Canada

**Keywords:** Monte Carlo dose calculations, heterogeneity corrections

## Abstract

Impact of the various kVp settings used during computed tomography (CT) simulation that provides data for heterogeneity corrected dose distribution calculations in patients undergoing external beam radiotherapy with either high‐energy photon or electron beams have been investigated. The change of the Hounsfield Unit (HU) values due to the influence of kVp settings and geometrical distribution of various tissue substitute materials has also been studied. The impact of various kVp settings and electron density (ED) distribution on the accuracy of dose calculation in high‐energy photon beams was found to be well within 2%. In the case of dose distributions obtained with a commercially available Monte Carlo dose calculation algorithm for electron beams, differences of more than 10% were observed for different geometrical setups and kVp settings. Dose differences for the electron beams are relatively small at shallow depths but increase with depth around lower isodose values.

PACS numbers: 87.57.Q‐, 87.55.D‐

## I. INTRODUCTION

Advances in modern computed tomography (CT) X‐ray imaging technology and its availability to radiotherapy departments led to widespread implementations of heterogeneity corrections to optimize dosimetry during treatment planning of patients undergoing radiotherapy treatments. A broad range of dose calculation algorithms have been developed (Monte Carlo, convolution/ superposition, pencil beam), providing improved dosimetric accuracy,^(^ ^1^ ^,^ ^2^ ^)^ but it has been shown^(3)^ that electron density must be taken into account in heterogeneity corrections. These algorithms rely on tissue‐dependent CT numbers acquired at the time of simulation used to estimate relative electron density of tissues to water using a Hounsfield Unit (HU) to electron density calibration curve.^(4)^ However, the HU is defined by the attenuation property of the absorbing materials and not only varies with the tissue type but also depends on the beam quality used for the CT scanning.^(5)^ In addition to the CT tube voltage (kVp), a number of factors have been found responsible for the variation of the HU for the very same type of materials (tissues): make and model of the CT scanner, field of view, scattering conditions and reconstruction algorithms.^(^ ^6^ ^,^ ^7^ ^,^ ^8^ ^)^ Consequently, if the HU value of the same material (having the very same electron density) will depend on the CT acquisition parameters, this may lead to dose calculation errors if the heterogeneity corrections are used when the treatment planning system converts the HU to electron density through a stored calibration curve.

The impact of electron density (ED) variation with CT acquisition parameters has been reported for high‐energy photon beams in the literature by investigating its dosimetric effect by using different heterogeneous phantoms.^(^ ^9^ ^,^ ^10^ ^)^ Cozzi at al.^(9)^ reported on the impact of an average HU to electron density calibration table provided by the manufacturer on computed doses when a custom calibration could not be included in the treatment planning system (TPS). While the applied voltage was the most relevant parameter leading to differences in the reconstructed Hounsfield numbers of about 300 units for high‐tissue densities, the maximum error determined on the computed monitor units per Gy was found to be about 2%, while being below 1% on average.^(9)^

Guan at al.^(10)^ investigated variations of dose per Monitor Unit (MU) versus different CT scanning parameters (kV, mAs) and different HU–ED curves for photon beams. They concluded that the dose/MU varies more with different kV and mAs for small field size 18 MV beams (smaller than 10 cm×10 cm) just beyond the high‐density bones. A smaller kV imaging beam quality will generate higher HU, which leads to a higher effective atomic number $\left(Z_{\text{eff}}\right)$ for given voxels. This will, in turn, result in a larger pair production fraction of the 18 MV treatment photon beam inside bone. For larger 18 MV field sizes (larger then 10 cm×10 cm), dose/MU variation with different kV is lesser because of the increased scatter. For 6 MV, the dose/MU varies less with different kV and beam size because of the dominant Compton scatter. Overall, Guan et al. concluded that the dose/MU from different HU–ED curves agrees to within 2%.

Depending on the CT simulator manufacturer and on the clinical practice, acquisition protocols might be optimized by the selection of the body section to be imaged, leading to different kVp setting for different body sites. On the other hand, HU to electron density (ED) calibration curves are not easily exchanged within commercially available treatment planning systems (TPS). Accordingly, in clinical practice one can either set up all the CT acquisition protocols to one beam quality (most commonly 120 kVp), or even if using various kVp settings (that will depend on the body site imaged), apply the very same HU to ED calibration curve (obtained at 120 kVp setting) during dose calculation using heterogeneity correction. The second approach is usually justified by the above‐cited findings that, even if using “unsuitable” HU to ED calibration curve, it will still provide an acceptable dose error of less than 2%.

While the impact of kVp settings on dose calculation accuracy for high‐energy photon beams is reported in the literature, in this work we investigate the impact of the various kVp settings used to scan the RMI heterogeneity phantom (RMI‐467, RMI, Gammex, Middleton, WI) on commercially available Monte Carlo based dose calculations (Eclipse, Varian Medical Systems, Palo Alto, CA) for radiotherapy electron beams. Since the real patient geometry may not be quite similar to distribution of tissue equivalent substitutes within the HU to ED calibration phantom, we also investigated the impact of the geometrical arrangement of electron density plugs within the RMI phantom on possible dose errors for both high‐energy electron and photon radiotherapy beams. (It should be noted that some other commercially available treatment planning systems use physical density instead of electron density for their heterogeneity corrections and Monte Carlo simulations.) Since the results reported in this work compare relative dose distribution values at selected points and, due to the fact that there is a fairly linear relation between physical and electron density,^†^ conclusions reached in this paper could be extended to the dose calculations using other treatment planning systems.

## II. MATERIALS AND METH ODS

### A.1 CT scans using different kVp settings and geometrical arrangements of ED plugs

To study the impact of different geometrical arrangements of various electron density plugs within heterogeneity phantom on HU to ED conversion curves, we used commercially available RMI‐467 heterogeneity phantom. Body of the phantom represents a solid water disk, 30 cm in diameter and 5 cm long with 16 holes distributed within two rings that can accommodate electron density plugs. These plugs are 1 inch in diameter and their electron density ranges from 0.28 to 1.69 relative to electron density of water.

**Figure 1:**
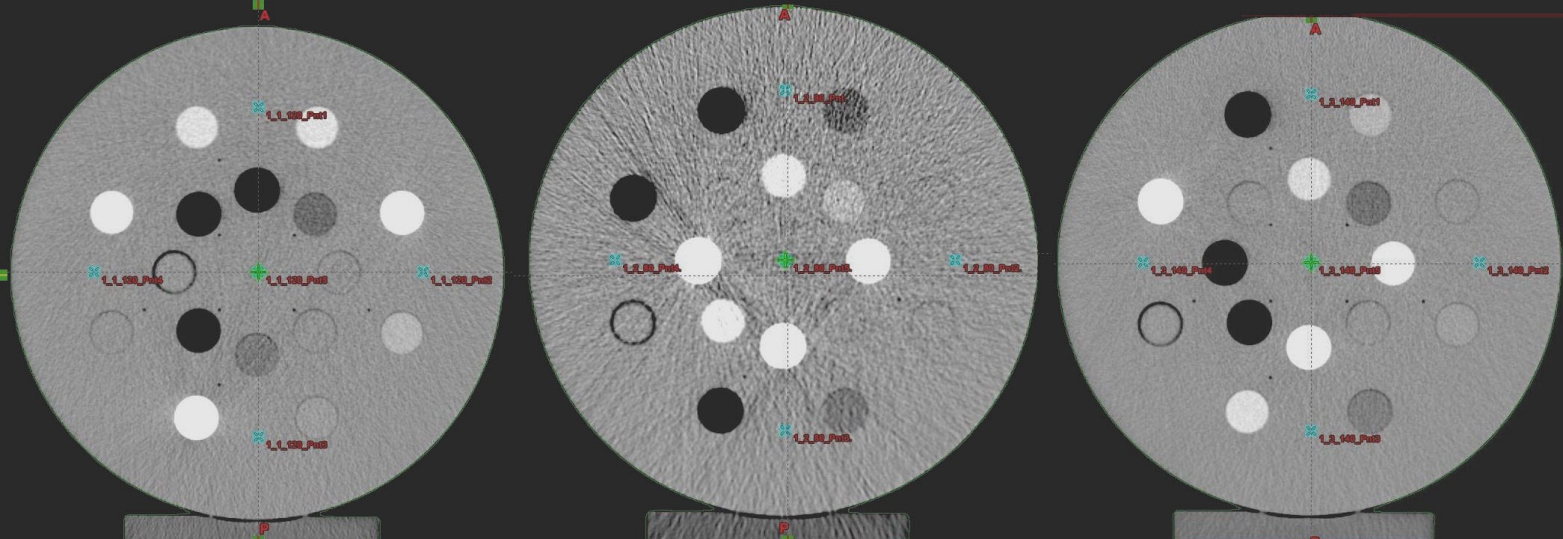
Three experimental setups of RMI‐467 heterogeneity phantom: Setup 1 (left), high electron density plugs within the outer ring; Setup 2 (middle), high electron density plugs within the inner ring; Setup 3 (right), electron density plugs randomly distributed.

Figure 1 represents three experimental setups of RMI‐467 heterogeneity phantom created for this study. In Setup 1, we placed high ED plugs within the outer ring of the phantom, while in Setup 2, high ED plugs were inserted within the inner ring. In Setup 3, ED plugs were randomly distributed and we call this setup a standard CT volume, as we assume that in clinical practice this would be the most commonly used geometrical arrangement of tissue ED plugs.

Each geometry setup was scanned at three kVp settings: 80 kVp, 120 kVp, and 140 kVp and 260 mAs on a GE High‐Speed CT simulator. CT slices with thickness of 3 mm were reconstructed with a standard convolution kernel with 512×512 pixels in size over 480 mm reconstruction diameter (0.9375 mm/pixel axial size). One HU to ED calibration curve was created and entered into Eclipse TPS, which corresponds to the standard CT volume. Dose distributions for both photon and electron beams calculated with this standard CT volume were used as a reference against which all the other dose calculations were compared. The HU values were scored for all nine CT volumes scanned in order to compare the impact of both kVp settings and geometrical arrangements on the change of the reconstructed CT numbers. Sampling of the HU values were carried out over square regions of interest (ROI) centered in the middle of each ED plug having 10×10 pixels in size.

#### A.2 Definitions of dose calculation volumes

A total of nine CT volumes for dose calculation has been created out of three geometrical setups scanned at three different kVp settings. Following the common clinical practice, CT volume obtained with Setup 3 at 120 kVp was assumed to be the standard CT volume to which all the data will be compared.

**Figure 2:**
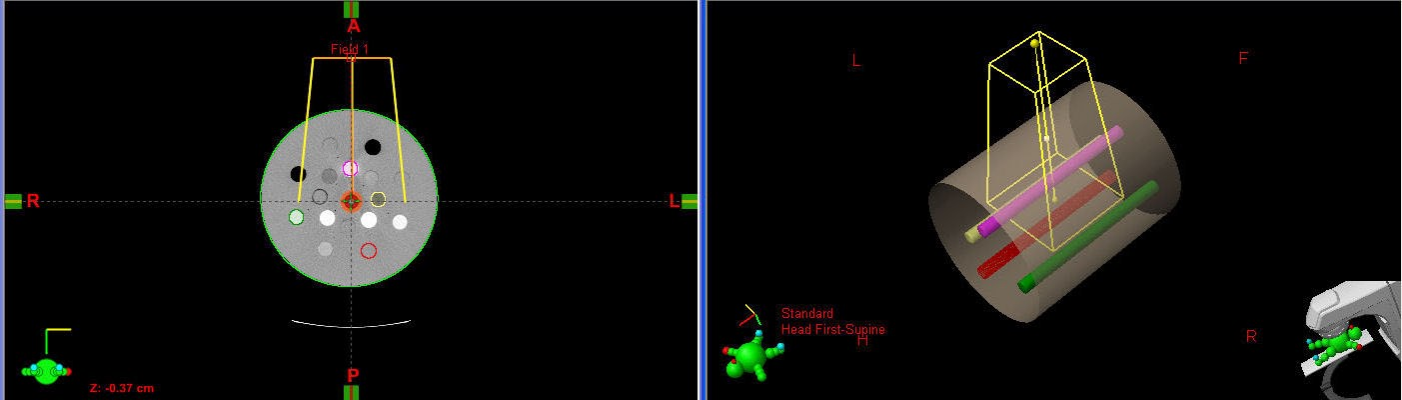
Extended CT phantom used for dose calculations with outline of the anterior beam: axial slice through the center of the phantom (left); three‐dimensional view (right) of the extended phantom to provide sufficient scattering conditions for the 20 cm×20 cm field size.

Since the original CT volume of the RMI‐467 phantom has only 5 cm longitudinal extension, CT volumes used for dose calculations were created within Selection workspace of the SomaVision (Varian Medical Systems, Palo Alto, CA) treatment simulation software by extrapolating the central CT slice of each CT volume by ± 15cm, resulting in a 30 cm long cylindrical volume (Fig. 2). Such a created volume represents a homogeneous longitudinal extension of the central slice which is commonly used to score HU values for the HU to ED calibration curve. Large CT volumes were created in order to provide full scattering conditions for the 20 cm×20 cm field sizes used for dose calculations. Owing to the cylindrical symmetry of such created volume, the choice of a slice thickness was arbitrary, and we decided to retain a clinical standard of 3 mm as previously indicated.

#### A.3 Dose algorithms used for the heterogeneous calculation

**Figure 3:**
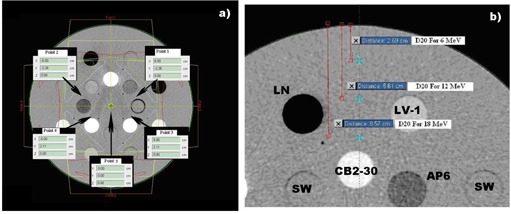
Points within phantoms for geometrical Setup 2 at which doses were scored: for photons (a), for electrons (b). Various tissue substitute plugs within RMI‐phantom for the electron beam simulations (right) are indicated: LN=450 Lung, LV−1= Liver, SW= solid water, CB2−30= Cortical Bone with $30\%{\text{CaCO}}_{3}$, AP6=Adipose.

Figure 3 represents dose calculation dose points which were used in comparing the effects of different CT volumes in this work. For the photon beams (Fig.3 (a)), dose was scored at five points for a standard four‐field box technique. One point (Point 5) was at the center of the phantom and isocenter of the four beams, while the other four points were chosen within the solid water medium but in close proximity to the heterogeneities. For electron beams (Fig.3 (b)) dose was scored at predefined percent depth dose points for an open beam and homogenous water phantom at D100, D90, D70, D50, and D20, along the central axis of the beam.

For photon dose calculations, pencil beam convolution algorithm (PBC ‐ 8614) was used in conjunction with modified Batho heterogeneity correction method and AAA calculation algorithms on Eclipse 8.6 (Varian Medical Systems) treatment planning system. Calculation grid size used was 2.5 mm and dose of 200 cGy was prescribed at the central point of the phantom (Fig.3 (a)), coinciding with the isocenter of the standard four‐field box plan. Beam weights were 1 for each beam and field size was set to 20 cm×20 cm. The dose in cGy per monitor unit was scored at each of the five points used in this comparison. Two clinical photon beams, 4 MV and 18 MV, of Varian 21 Ex linear accelerator were used for calculations.

For the electron beam dose calculations, we used the electron Monte Carlo (eMC Version: 8.6.14) calculation algorithm on the same Eclipse 8.6 treatment planning system. We used a grid size of 2.5 mm and 3D Gaussian (medium) smoothing level.^(11)^ Random number generator seed was set to 39916801, with an accuracy set to 2%. A single electron beam defined with 15 cm×15 cm cone was set in a source to surface distance (SSD) setup of 100 cm at the top of the RMI phantom. Three clinical electron beams, 6 MeV, 12 MeV and 18 MeV, of Varian 21 Ex linear accelerator were used for calculations. As in the case of photon beams, a 200 cGy prescription dose was prescribed at the depth of maximum dose $\left(z_{\text{max}}\right)$ for every particular beam quality.

For both photon and electron beams, we calculated percentage dose differences at every dose calculation point for a given CT volume (kVp setting and geometry), as compared to the dose calculated for the standard CT volume (120 kVp, Setup 3). At first, the actual (or “wrong”) dose values had been calculated for all nine CT volumes using measured HU within every CT volume to determine pixel‐by‐pixel ED from the calibration curve determined using the standard CT volume (120 kVp, Setup 3). Then, another set of accurate (or “true”) dose distributions were calculated for all eight CT volumes (excluding the standard CT volume). However, this time the actual known ED values for every outlined volume from the manufacturer's ED phantom specification sheet was assigned, thereby ignoring the actual HU values. In order to calculate the actual dose error between prescribed dose (based on standard CT volume) and dose delivered to the patient/phantom (based on the “true” CT volume), we used the dose per MU quantity instead of dose itself.

## III. RESULTS

**Figure 4:**
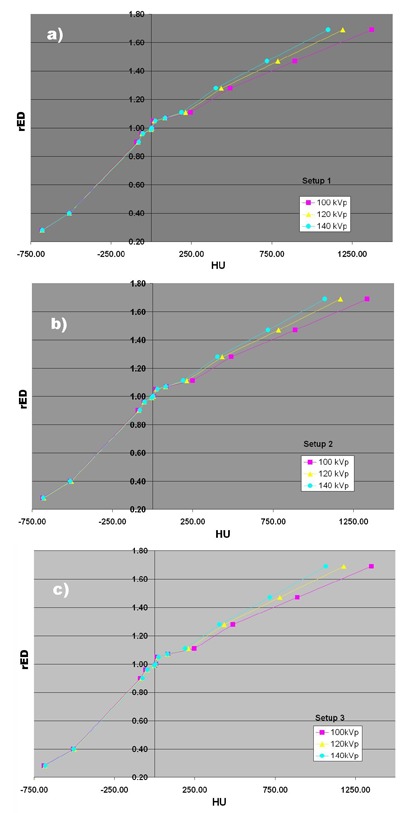
Relative electron density changes as a function of reconstructed HU values for various kVp tube settings and geometrical setups simulated in this work: a) Setup 1, b) Setup 2, c) Setup 3.

It is apparent from Fig. 1 that geometrical arrangement of electron density plugs can affect the image quality as a consequence of the change in the reconstructed HU values (window and level settings have been set to the same values for all three axial scans). Figure 4 represents changes in relative electron densities as a function of the reconstructed HU values for various kVp tube settings and geometrical setups investigated in this work. Table 1 summarizes relative changes of $\left(\mu /\mu_{\text{wat}}\right)$ ratios for different ED tissue substitutes in three geometrical setups at three kVp X‐ray tube settings as estimated from the sampled HU values, given in Fig. 4, using the following mathematical expression:

(1) $$\left(\frac{\mu }{\mu_{\text{wat}}}\right)=\left(\frac{\text{HU}}{1000}\right)+1$$

representing the actual definition of the Hounsfield Unit. The first column in Table 1 lists relative electron densities to water, hereinafter referred to as rED. Results presented in Table 1 indicate that various geometrical arrangements of rED plugs have an impact on the reconstructed HU values. The largest variation in $\left(\mu /\mu_{\text{wat}}\right)$ is observed for high Z materials, being positive at lower and negative at higher kVp settings compared to the standard CT volume.

**Table 1:** Relative change of $\left(\mu /\mu_{\text{wat}}\right)$ ratios for different rED tissue substitutes in three geometrical setups at three kVp X‐ray tube settings as estimated from the sampled HU values given in Fig. 4.

	*Setup 1*			*Setup 2*			*Setup 3*	
*rED*	*80 kVp*	*120 kVp*	*140 kVp*	*80 kVp*	*120 kVp*	*140 kVp*	*80 kVp*	*140 kVp*
0.28	5.7%	0.4%	0.9%	6.5%	1.5%	0.8%	2.5%	0.7%
0.40	1.7%	−1.8%	−1.8%	1.1%	−1.2%	−2.3%	3.6%	−0.6%
0.90	−2.6%	−0.2%	0.4%	−2.3%	−0.6%	0.2%	−1.7%	0.7%
0.96	−3.2%	−1.2%	−0.4%	−1.4%	−0.5%	0.0%	−1.6%	0.3%
0.99	−0.5%	0.1%	0.1%	0.2%	−0.3%	−0.3%	0.2%	0.1%
1.00	−0.3%	−0.3%	−0.3%	0.2%	−0.2%	−0.3%	0.1%	0.0%
1.05	−2.2%	−0.3%	0.7%	−1.5%	0.2%	1.0%	−1.7%	0.5%
1.07	1.0%	0.5%	0.6%	0.2%	0.1%	0.2%	0.0%	0.1%
1.11	6.7%	0.2%	−1.8%	7.0%	0.1%	−1.6%	6.5%	−1.7%
1.28	8.4%	0.1%	−2.2%	7.6%	0.0%	−2.1%	7.3%	−2.2%
1.47	13.8%	0.4%	−3.3%	12.0%	0.2%	−3.5%	11.7%	−3.4%
1.69	17.3%	0.7%	−3.5%	14.5%	−0.4%	−4.8%	17.2%	−5.1%

Table 2 summarizes percentage differences of dose per MU for three different geometrical setups and three X‐ray tube kVp settings. Dose differences were calculated with respect to the standard CT volume (Setup 3 at 120 kVp) for two megavoltage photon beams using both modified Batho and AAA dose calculation methods. Percentage dose differences are listed at five dose calculation points, defined above for photon beam calculations. Values listed in Table 2 are direct dosimetric consequence of the variation of HU values indicated in Fig. 4. For each setup, these values represent dose differences between the doses calculated using calibration curve obtained with standard CT volume and the true dose distribution obtained by assigning the actual rEDs to tissue substitute plugs, as follows:

(2) $$\frac{\Delta D}{D}=\frac{D\left(rED=f(HU)\right)-D(rED_{true})}{D(rED_{true})}$$

**Table 2:** Percentage differences of dose per MU for three different geometrical setups and three X‐ray tube kVp settings calculated with respect to the standard CT volume S3 (Setup 3 at 120 kVp) for two megavoltage photon beams using modified Batho and AAA dose calculation methods.

	*Setup 1*			*Setup 2*			*Setup 3*	
*Points*	*80 kVp*	*120 kVp*	*140 kVp*	*80 kVp*	*120 kVp*	*140 kVp*	*80 kVp*	*140 kVp*
4 MV ‐ Mod. Batho								
1	0.0%	0.0%	0.2%	−0.1%	0.1%	−0.6%	−0.3%	0.0%
2	0.0%	0.1%	0.2%	−0.4%	0.0%	0.3%	−0.2%	0.0%
3	0.2%	0.0%	0.0%	−0.1%	0.2%	1.3%	0.0%	−0.1%
4	−0.6%	−0.1%	0.0%	−0.6%	0.1%	0.4%	−0.3%	0.0%
5	0.0%	0.0%	0.0%	−0.6%	0.0%	0.6%	−1.0%	−0.1%
4 MV – AAA								
1	−0.3%	0.0%	0.0%	−0.2%	0.0%	0.0%	−0.1%	0.0%
2	−0.4%	0.0%	0.1%	−0.8%	−0.4%	−0.5%	−0.2%	0.0%
3	−0.2%	0.0%	−0.1%	−0.2%	0.1%	0.1%	−0.1%	−0.1%
4	−0.7%	0.0%	0.1%	−0.7%	−0.2%	−0.3%	0.0%	0.0%
5	0.0%	0.0%	0.0%	−0.9%	−0.3%	−0.3%	−0.6%	0.0%
18 MV ‐ Mod. Batho								
1	−0.1%	0.0%	0.0%	−1.0%	−0.4%	−1.1%	−0.5%	0.0%
2	0.0%	0.1%	0.1%	−1.2%	−0.4%	−0.5%	−0.4%	0.0%
3	0.1%	0.0%	0.0%	−1.1%	−0.4%	−0.1%	−0.3%	0.0%
4	−0.4%	−0.1%	0.0%	−1.2%	−0.4%	−0.5%	−0.5%	0.0%
5	0.0%	0.0%	0.0%	−1.3%	−0.4%	−0.4%	−0.9%	0.0%
18 MV – AAA								
1	−0.1%	0.0%	0.0%	−0.8%	0.0%	0.0%	−0.1%	0.0%
2	−0.2%	0.0%	0.1%	−1.1%	0.0%	−0.1%	−0.2%	−0.1%
3	−0.1%	0.0%	0.0%	−0.8%	0.0%	−0.1%	−0.2%	−0.2%
4	−0.4%	−0.1%	0.0%	−1.0%	0.0%	0.0%	−0.2%	−0.1%
5	0.0%	0.0%	0.0%	−1.3%	0.0%	0.0%	−0.4%	0.0%

Table 3 represents percentage dose per MU differences for three different geometrical setups and three X‐ray tube kVp settings calculated with respect to the standard CT volume (Setup 3 at 120 kVp) for three megavoltage electron beams using electron Monte Carlo dose calculation algorithm. Percentage dose differences are listed at five points, defined above for electron beam calculations. As in Table 2, columns labeled by the kVp settings in Table 3 are direct dosimetric consequence of the variation of the HU values indicated in Fig. 4 and are calculated using Eq. (2).

**Table 3:** Percentage difference of dose per MU for three different geometrical setups and three X‐ray tube kVp settings calculated with respect to the standard CT volume S3 (Setup 3 at 120 kVp) for three electron beams.

	*Setup 1*			*Setup 2*			*Setup 3*	
*Points*	*80 kVp*	*120 kVp*	*140 kVp*	*80 kVp*	*120 kVp*	*140 kVp*	*80 kVp*	*140 kVp*
				6 MeV				
D100	−0.6%	0.1%	−0.2%	0.0%	0.0%	−0.2%	−0.3%	−0.7%
D90	2.7%	0.0%	0.1%	1.3%	−0.2%	2.2%	2.2%	2.9%
D70	4.8%	0.0%	0.3%	2.2%	−0.7%	4.2%	5.1%	6.3%
D50	7.4%	−0.1%	0.1%	2.8%	−0.8%	5.6%	7.0%	8.6%
D20	12.5%	−0.5%	−0.7%	4.2%	−2.5%	8.9%	12.9%	15.6%
				12 MeV				
D100	0.6%	0.0%	0.5%	0.0%	−0.1%	0.5%	−0.2%	0.5%
D90	1.7%	0.0%	0.7%	0.1%	−0.7%	1.2%	1.3%	2.0%
D70	2.2%	−0.3%	−0.1%	0.4%	−0.7%	2.2%	2.5%	2.9%
D50	3.4%	−0.6%	−0.6%	0.7%	−0.6%	3.8%	3.4%	4.4%
D20	7.2%	−1.0%	−0.5%	1.5%	−2.1%	7.4%	6.0%	8.8%
				18 MeV				
D100	0.8%	0.8%	0.0%	−0.1%	0.8%	0.2%	−0.4%	0.4%
D90	−1.7%	0.3%	0.1%	−0.3%	−1.0%	−5.7%	−2.0%	−13.4%
D70	−1.3%	0.3%	0.3%	−0.2%	−1.4%	−5.0%	−1.4%	−12.0%
D50	0.0%	0.5%	1.0%	6.6%	−1.2%	−3.2%	−1.1%	−11.5%
D20	0.0%	0.0%	0.0%	0.0%	0.0%	0.0%	0.0%	0.0%

## IV. DISCUSSION

Our results for the standard CT volume configuration (Setup 3 in Table 1) are in good agreement with previously reported HU variations as a function of the CT scanning technique employed^(^ ^9^ ^,^ ^10^ ^)^ reporting the maximum variation for high Z tissue substitutes. The same trend appears to propagate through the various geometrical setups studied in this work, as well. Reason for the high Z materials having the largest impact on the observed $\left(\mu /\mu_{\text{wat}}\right)$ ratios could be explained by the nonuniform beam hardening effect of the scanning beam passing through various materials. Commercial reconstruction software includes a beam hardening correction routines, which are based on homogeneous water phantom data. This, in turn, explains why the variations in the $\left(\mu /\mu_{\text{wat}}\right)$ ratios around water‐similar substitutes are relatively small. Results presented in Table 1 indicate relatively large impact of the beam quality on $\left(\mu /\mu_{\text{wat}}\right)$ ratio variation within the same geometrical setup. On the other hand, when the $\left(\mu /\mu_{\text{wat}}\right)$ ratio variation is compared within the same beam quality over the three geometrical setups, the variation appears to be relatively small. Actually, the latter variations appear to be smaller than the actual relative standard deviations of the sampled $\left(\mu /\mu_{\text{wat}}\right)$ ratio values, indicating that the observed variations will be primarily affected by the beam quality rather than by the actual scanning geometry.

Although the percentage change of the $\left(\mu /\mu_{\text{wat}}\right)$ ratios can be as large as almost 20% for high Z tissue substitutes (Table 1), the more important question is: What the impact of this change on dose calculations is when tissue heterogeneity corrections are applied? Dose differences for high‐energy photon beams and standard CT volume (Setup 3) given in Table 2 indicate that even with varying the kVp setting, change in dose is well within 1%, as suggested by Cozzi et al.^(9)^ The very same trend for high‐energy photon beams is observed for the two additional geometrical setups. Nevertheless, results shown in Table 2 suggest that when both geometrical arrangement of electron density plugs and variation in kVp settings are combined, the overall percentage dose difference drops to within 1%, as compared to the standard CT volume (Setup 3 at 120 kVp setting). This conclusion is in agreement with commonly adopted clinical practice that variation in kVp setting during the CT simulation does not introduce significant error in heterogeneity based dose calculations for high‐energy photon beams. Our results extend this conclusion to all anatomical sites, regardless of the geometrical variation of electron densities when compared to the standard CT volume (Setup 3 at 120 kVp) used to create HU to rED calibration curve. Our results also indicate that, despite the fact that AAA algorithm might be considered more accurate for dose calculations in high‐energy photon beams, the kVp settings and geometrical arrangements of ED do not differ significantly when compared to the PBC algorithm. Improvement in dose accuracy is better for the 4 MV photon beam quality, while the dose differences for the 18 MV beam quality appear to be identical for both AAA and modified Batho method. It is also of note that geometrical Setup 2 imaged with 80 kVp beam quality shows the largest variation in calculated doses. However, in addition to the particular geometry (high Z materials concentrated within the central region of the phantom), relatively large image noise (observed in Fig. 1 middle) could be also responsible for the largest observed variation in dose for 18 MV photon beam.

On the other hand, results presented in Table 3 suggest that in electron beams, even within Setup 3, difference in kVp setting may result in dose differences of up to 16% at low‐dose levels for 140 kVp setting for lowest electron beam quality (6 MeV) studied. These errors in low‐dose regions may have significant clinical impact on treatment planning of electron boost fields (for ENT, for example), whereas the critical structures to be protected beyond the target may receive significantly higher dose (more than 10%) than anticipated. The large observed dose differences for electron beam at low doses are expected due to the fact that at low electron energies the collisional stopping power for electrons in all tissue‐like materials increases rapidly and becomes more sensitive to even small tissue composition differences. While the observed dose errors increase with depth in low electron beams, it appears that in the highest (18 MeV) energy electron beam, dose errors actually decrease with depth. One has to keep in mind that the first reported point for the 18 MeV beam is at 3 cm depth, which is in turn D20 for the 6 MeV beam. Also, the side‐scattering is a dominant effect in dose deposition by the electron beams. On the other hand, backscattering of the electron beams decreases by increasing the electron energy, and this further explains the fact that we did not register any dose difference at depth of D20 for the 18 MeV electron beam.

The actual dose errors reported in Table 3 are due to the use of the inappropriate HU to rED conversion curve. Our results suggest that the effect of kVp settings for CT simulation is significant and cannot be ignored for high‐energy electron beam dose calculations.

## V. CONCLUSIONS

We report on the impact of the various kVp settings used during CT simulation, which provides data for accurate heterogeneity corrected dose distributions in patients undergoing external beam radiotherapy with either high‐energy photon or electron beams. Since distribution of various electron density materials may vary throughout different anatomical sites, we also investigated the impact of the geometrical arrangement of various known tissue substitutes on both reconstructed HU values, and calculated dose distributions if only one standard HU to rED calibration curve is used.

We found that both kVp settings, as well as geometric distribution of various materials, lead to significant change of the HU values, the largest being for high Z materials and lowest kVp setting used for the CT scanning. We also observed a small change in the HU value with change in the geometrical arrangement of the various electron density materials when the kVp setting is kept the same. Impact of various kVp settings and rED distribution on the accuracy of dose calculation in high‐energy photon beams was found to be well within 2%, in accordance to previously published data and common clinical practice of using the standard HU to rED calibration curve created by a 120 kVp CT acquisition technique. However, in the case of dose distributions obtained with commercially available Monte Carlo dose calculation algorithm for electron beams, differences of more than 10% between different geometrical setups and kVp settings were observed. While dose differences for the electron beams are relatively small at shallow depths (around D100), they increase with depth in the vicinity of lower isodose values.

## Footnotes

† Gammex 467 Tissue Characterization Phantom is available from: http://www.GAMMEX.COM/ACE‐IMAGES/ONCOLOGY/RO_467.PDF
